# Computational study on the transmission of the SARS-CoV-2 virus through aerosol in an elevator cabin: Effect of the ventilation system

**DOI:** 10.1063/5.0068244

**Published:** 2021-10-25

**Authors:** N. N. Peng, K. W. Chow, C. H. Liu

**Affiliations:** Department of Mechanical Engineering, University of Hong Kong, Pokfulam, Hong Kong

## Abstract

Aerosol transmission is now well-established as a route in the spread of the SARS-CoV-2 virus. Factors influencing the transport of virus-laden particles in an elevator cabin are investigated computationally and include human respiratory events, locations of the infected person(s), and the ventilation system (ventilation mode, ventilation capacity, and vent schemes). “Breath,” “cough,” and “sneeze” are defined quantitatively by the fluid jet velocities and particle sizes. For natural ventilation, most particles exhaled by sneezing and coughing tend to deposit on surfaces quickly, but aerosol generated by breathing will remain suspended in the air longer. For forced ventilation, motions of particles under different ventilation capacities are compared. Larger particles otherwise deposited readily on solid surfaces may be slowed down by airflow. Air currents also accelerate the motions of smaller particles, facilitating the subsequent deposition of micrometer or sub-micrometer particles. Locations of the infected person(s) lead to different spreading scenarios due to the distinctive motions of the particles generated by the various respiratory events. Sneeze particles will likely contaminate the person in front of the infected passenger only. Cough particles will increase the risk of all the people around the injector. Breath particles tend to spread throughout the confined environment. An optimized vent scheme is introduced and can reduce particles suspended in the air by up to 80% as compared with commonly used schemes. The purification function of this vent model is robust to various positions of the infected passenger.

## I. INTRODUCTION

The ongoing pandemic arising from the SARS-CoV-2 virus has resulted in large mortality/morbidity rates,^1^ enormous economic losses,^2^ and severe disruption in modern lifestyle.^3,4^ The main agents responsible for spreading the SARS-CoV-2 virus are typically virus-laden aerosols formed and exhaled through the mouth or nose of the infected person(s) by breathing, talking, coughing, or sneezing.^5,6^ The diameter of aerosols generated by human expiratory events varies from sub-micrometer to hundreds of micrometers. Large droplets generally deposit on surfaces quickly, while small aerosols efficiently disperse in the air.^7^ Hence, virus loaded on particles will spread out widely in the medium.^8,9^ The transport of particles then causes three primary routes of infection, namely, saliva directly spattering onto the mouth or nose, physical contact with deposited droplets, and inhalation of aerosol flow generated by infected people.^10^ The last route, i.e., airborne transmission, has been identified as the dominant mechanism in the spreading of the coronavirus.^11^

In urban areas, daily activities in confined spaces, such as buses, trains, buildings, and elevators, are inevitable. For example, there are almost 65 000 elevators in Hong Kong, a metropolitan area with 7.4 million residents.^12^ Several coronavirus disease outbreaks in confined spaces indicate that indoor transmission of the virus is efficient and may be associated with the ventilation system.^13–16^ The transmission in an indoor environment poses a larger risk than one for outdoor, possibly due to the longer exposure times and the limited airflow found in an indoor setting.^17,18^ To minimize the risk of infection indoor, it is crucial to investigate the dynamics of airflows and aerosol transport in an enclosed space, and the effect of the ventilation system.

After the outbreak of the COVID-19 pandemic, intensive efforts have been invested to study the mechanism of indoor virus transmission. Aerosol transport in a realistic classroom was investigated. The influence of particle size, aerosol source location, glass barriers, and windows on the transmission and deposition of particles is elucidated.^19^ In an earlier study on particle transport in elevators, the placement and design of the air purifier and ventilation systems are found to significantly affect droplet dispersion.^20,21^ The present effort reinforces these existing works in demonstrating the effectiveness of computational fluid dynamics (CFD) in modeling airflow and the spread of the virus. The roles of the ventilation system are highlighted. The importance of vent schemes is emphasized. With proper design of inlet/outlet positions, the risk of infection can actually be mitigated. Certain simplifying assumptions, e.g., turbulence modeling and range of particle sizes, have been made. These computational predictions should be verified experimentally in the future whenever possible. From the findings of these studies, flow dynamics analysis using CFD is recommended in the design of similar systems.

A series of experiments on the dispersal of viral aerosols along a poorly ventilated corridor has also been conducted.^22^ For transportation vehicles, the flow that carries aerosols in buses is predominantly controlled by the ventilation system.^23,24^ In a general study on particle transmission in an elevator, a small classroom, and a supermarket, inappropriate design of ventilation systems is shown to drastically degrade the efficiency of particle removal, creating hot spots with tremendously higher risks.^25^

The aforementioned studies highlight the importance of the ventilation system in the spreading of the virus in confined spaces. However, differences caused by human activities and vent schemes have not been properly addressed. For a functioning elevator in a commercial or residential building, combined actions of the ventilation system, passengers entering and leaving, and frequent opening and closing of the door will induce very complex flow patterns inside the lift cabin. Reducing the risk of viral respiratory disease infection in elevator cabins will be an important practical problem for fluid dynamicists.^20,21^ A pragmatic approach in mitigating respiratory infections is achieved by improving air circulation. We shall utilize OpenFOAM, an open-source CFD library, to examine the flow parameters involved. The physical principle adopted is the Eulerian–Lagrangian approach, one of the widely used multi-phase computational schemes, for simulating the transport of respiratory fluid particles. Three factors,

• human respiratory events,
• locations of the infected person(s), and
• ventilation system (which will be further classified into (a) ventilation mode/capacity, and (b) vent schemes),

are investigated to determine the relationship between ventilation system design and the control of the aerosol transmission. Finally, an optimized vent scheme will be designed, and its performance in eliminating suspended particles will be assessed.

## II. METHODS

### A. Computational domain and mesh

**FIG. 1.**
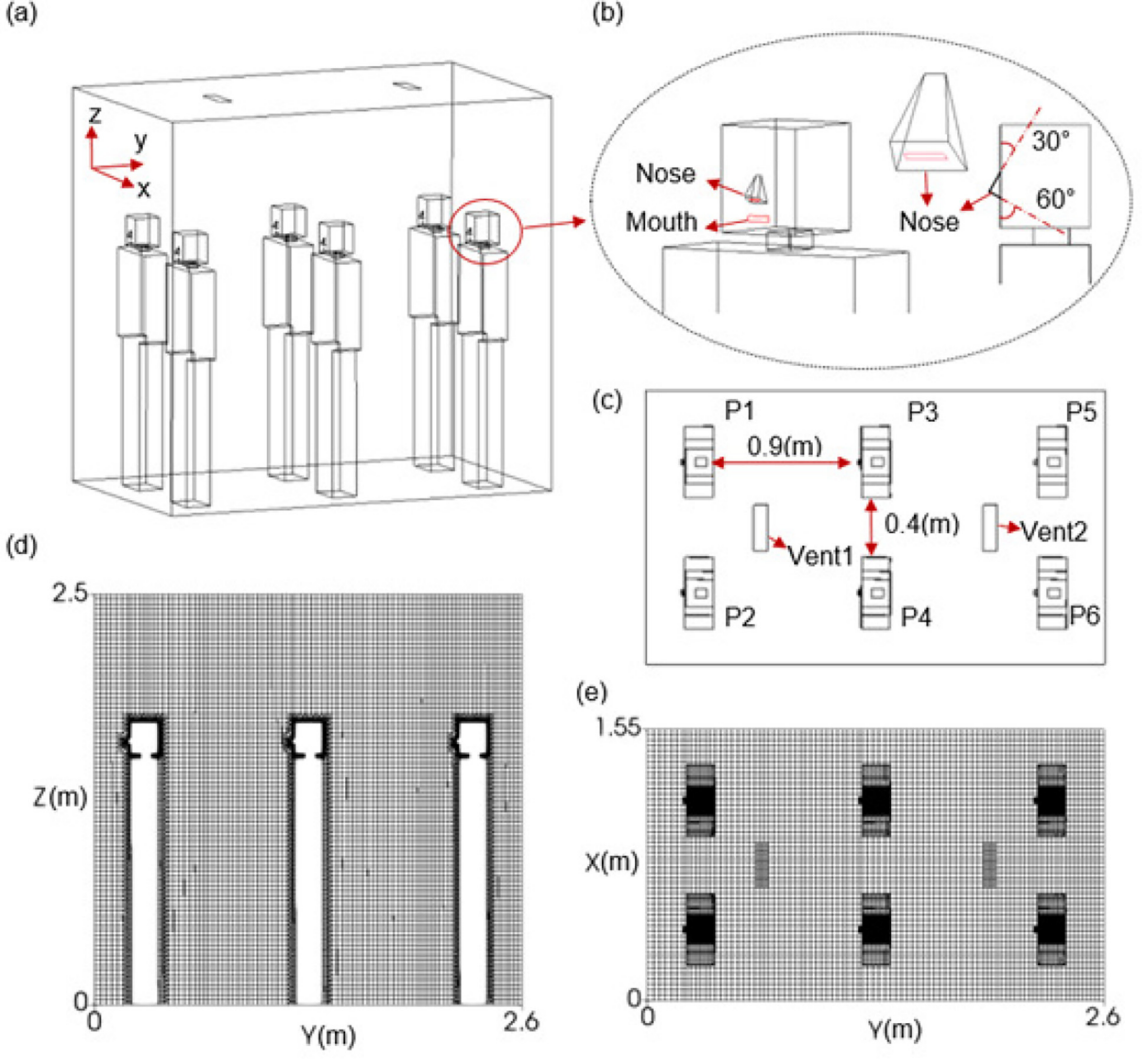
(a) 3D modeling of the elevator, (b) enlarged view of the head, (c) configuration of the standing passengers, and (d) and (e) illustration of the computational mesh.

A three-dimensional (3D) model of an elevator with six passengers is developed for mimicking realistic scenarios in daily life (Fig. 1). The dimensions of the lift cabin are 1.55 m wide, 2.6 m long, and 2.5 m high. Two or more vents on the ceiling or sidewalls of the cabin constitute the inlet of supply air and the outlet of return air for the air conditioning system. Inside the cabin, six passengers are positioned along two lines 0.4 m apart. The passengers in each row are equally spaced with a distance of 0.9 m. The 3D configuration of each passenger consists of

(a) a model of 1.7 m high and
(b) two rectangular blocks representing the head and the body [Fig. 1(b)].

For the “head” of the model, the feature of the mouth is captured by a rectangular surface with an area of 0.04 × 0.01 m^2^. The nose is taken as a trapezoidal wedge which adheres to the front surface of the head cuboid. The nostrils are also formed by a rectangle with an area of 0.02 × 0.005 m^2^ at the bottom of the wedge. The angle between the bottom surface and the positive z-axis (vertical) is 60°.^26^

For minimizing memory and CPU time, a Hex-dominant mesh is generated using the *snappyHexMesh* utility in *OpenFOAM*, with a background hexahedral mesh discretizing the lift cabin using *ICEM CFD*. Independent surfaces are defined in the model for proper mesh refinement and for tracking the aerosol deposition on each wall and passenger. Approximately 650 000 computational cells are used. The grids near the surfaces of the passengers are refined with a minimum cell size of 1.75 mm, and the maximum cell size of the whole computational domain is 28 mm. The size and quality of the mesh have been tested for their capability to resolve the turbulent flows and particle transport adequately (Sec. II E 3).

### B. Respiratory flows and particles

One important mechanism in the spread of the SARS-CoV-2 virus is the respiratory route, where the infected person exhales small droplets carrying the virus by coughing, sneezing, singing, talking, or breathing. The properties of air streams laden with particles with pathogens are complex. For the purpose of quantifying the dynamics, we define these respiratory events using the following fluid dynamics parameters:

• Human “breath” emits small particles at micrometer and sub-micrometer scales with relatively slow speed, with a maximum velocity of around 1 m/s.^7,25^
• “Cough” ejects larger particles at micrometer-scale with a higher speed of up to 10 m/s.^27–29^
• “Sneeze” exhales still larger particles with diameters of 10–300 mm at even higher transient speed, with a maximum value of over 15 m/s.^30,31^

**FIG. 2.**
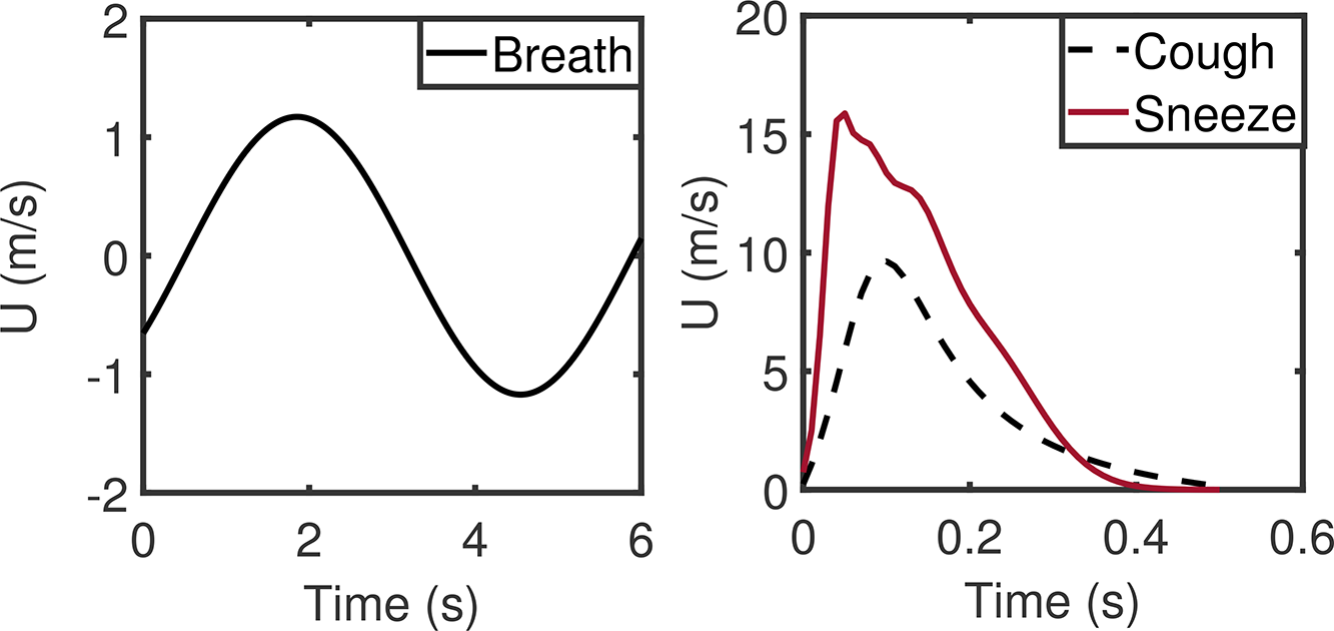
Time traces of airflow velocity.

**FIG. 3.**
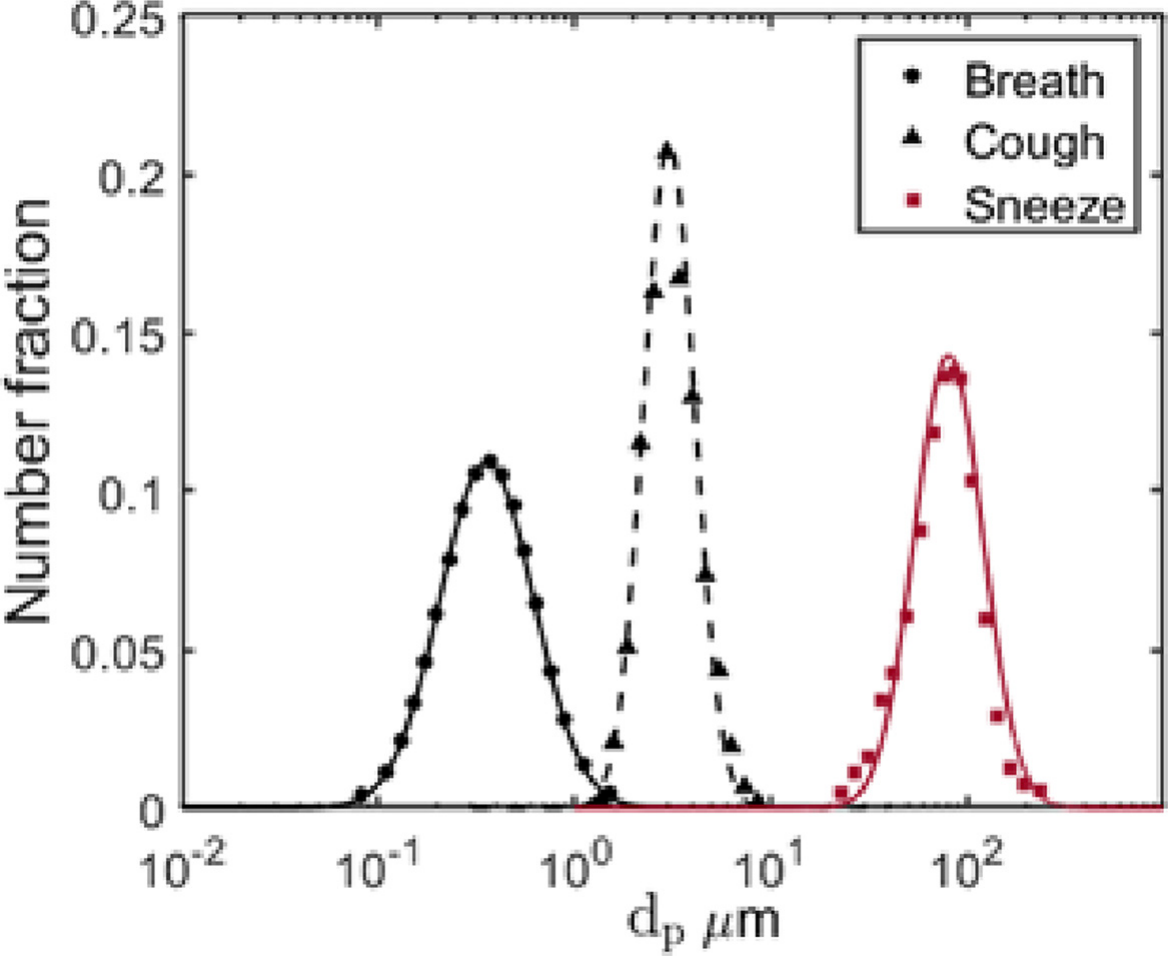
Particle size distribution.

The time traces of airflow velocity induced by breathing, coughing, and sneezing are shown in Fig. 2. The profile of breath is persistent and near-sinusoidal with a period of about 5.7 s. On the contrary, both cough and sneeze are jet flows localized in time which last for less than 0.5 s. The size distributions of particles emitted by these three kinds of respiratory events approximately follow the lognormal distribution (Fig. 3). Although some studies^28,29^ have suggested a double-peaked droplet size distribution for both coughing and sneezing, only the first peak with smaller particles is considered since the other peak with larger particles contributes less to the transmission of the virus through air. The number of particles exuded by coughing or sneezing varies considerably.^32–34^ In this study, 3500 particles per cough and 11 700 particles per sneeze are ejected. Breath is a periodic behavior that exhales around 1600 particles every respiratory cycle.^25^

### C. Ventilation system

One natural mode of ventilation and two forced modes, with ventilation capacities of 110 and 560 m^3^/h, are chosen for this study. The volume of the lift cabin is 9.75 m^3^, and thus the air changes per hour (ACH) are 11.28 and 57.44 for the two forced modes.

**FIG. 4.**
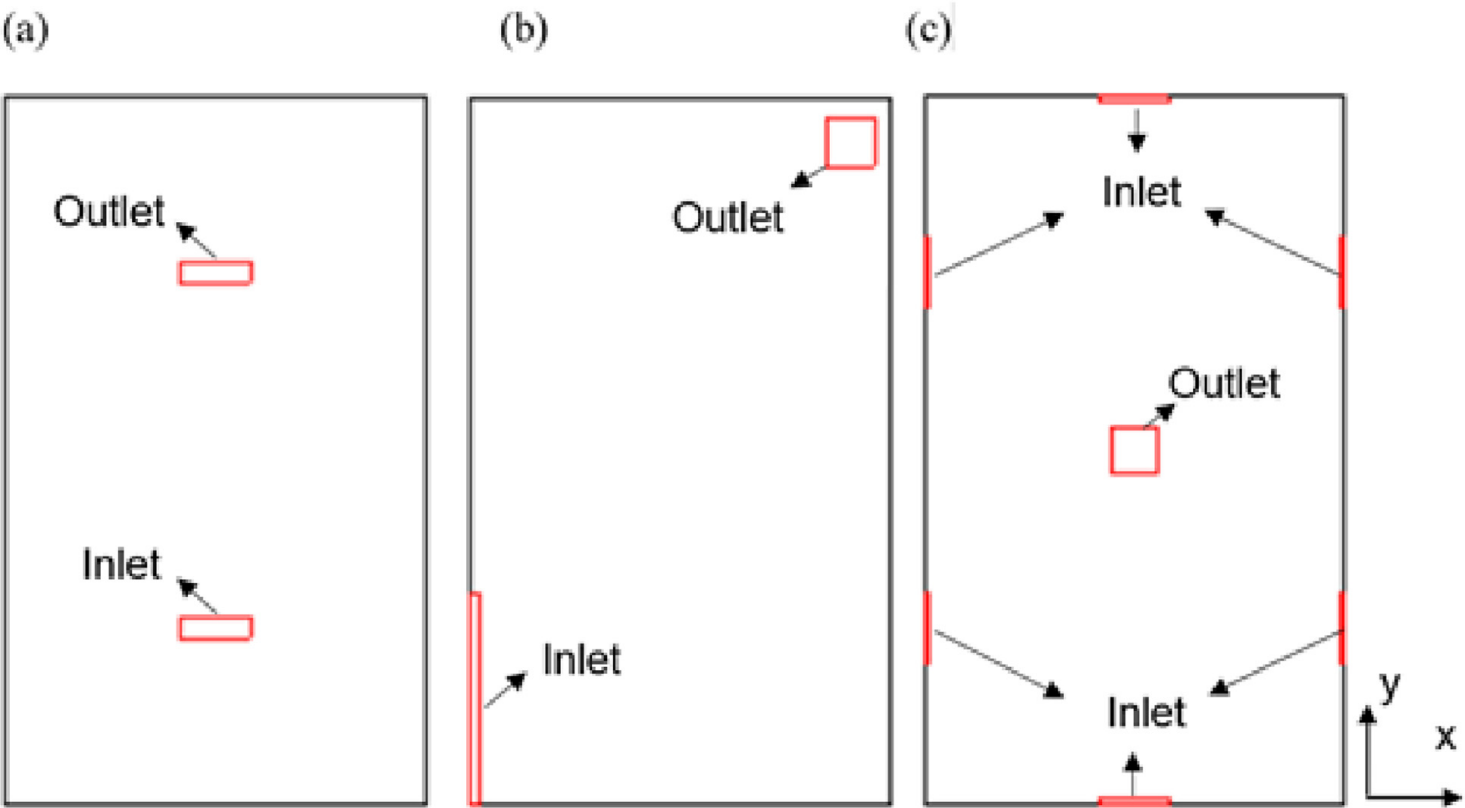
Three ventilation schemes with vents on the ceiling only (top view): (a) vent model 1, (b) vent model 2, and (c) vent model 3.

**FIG. 5.**
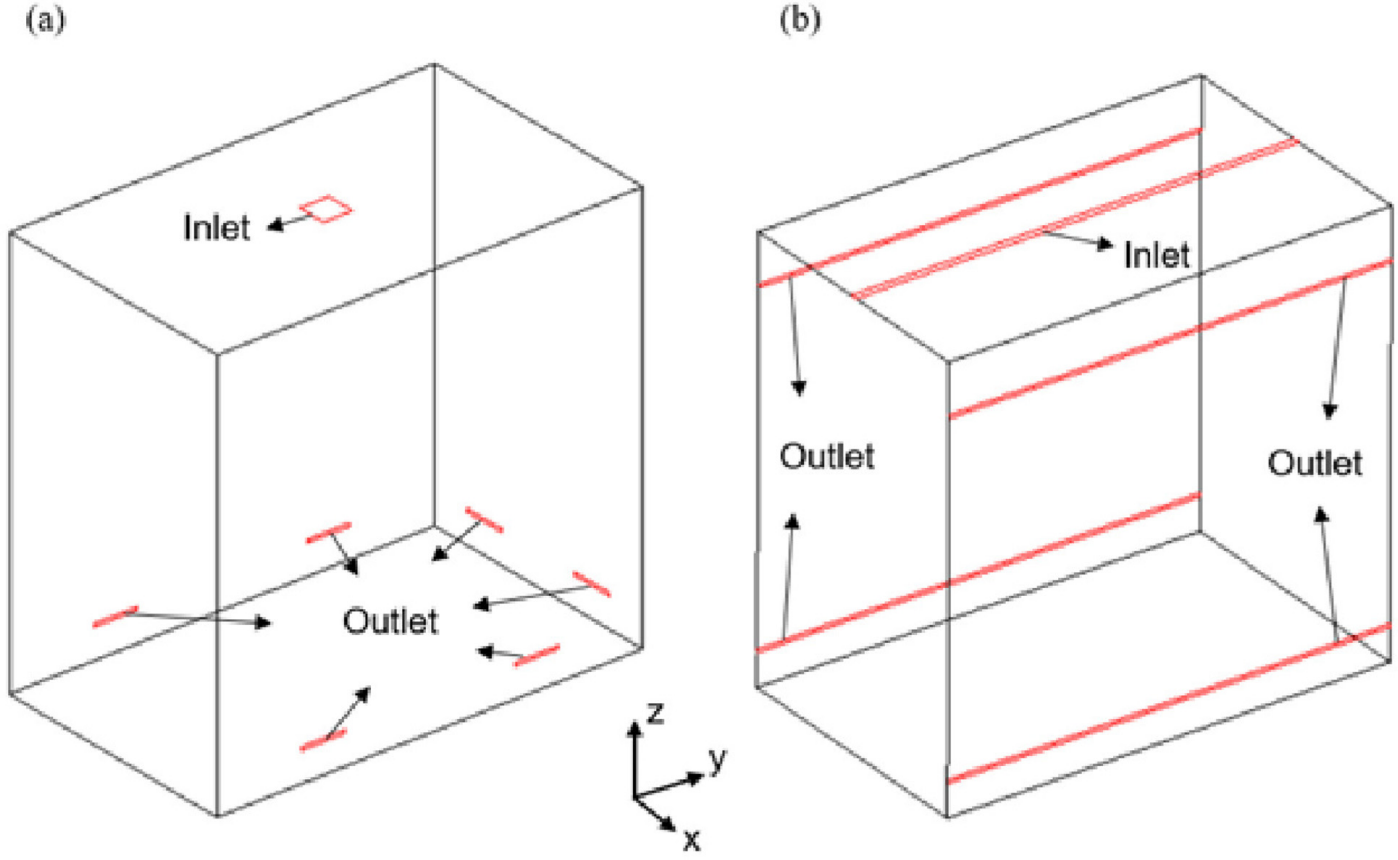
Two ventilation schemes with vents on both the ceiling and the sidewalls: (a) vent model 4 and (b) vent model 5.

The air vents of a lift usually are slatted openings on sidewalls or ceiling. In this work, the performances of five vent models are analyzed. Three typical schemes of ceiling vent design^35,36^ (Fig. 4) and two vent models that adopt both ceiling and sidewall vents^37^ (Fig. 5) will be selected. The area of the inlet vent of the first four vent models is assumed to be equal to the outlet vent area, which is 0.0312 m^2^. The dimensions and flow velocities (with ACH being 57.44) of the vents are listed in Table I. In the natural ventilation cases, both the vents of the inlet and outlet are treated with a pressure boundary condition (BC), while in the forced ventilation cases, in which the ACH is 11.28 or 57.44, the velocity at the inlet is 0.98 or 4.99 m/s. For the optimized vent scheme, vent model 5, the inlet area and outlet area have been expanded to 0.104 m^2^ and 0.208 m^2^, respectively. Only the scenario with the ACH being 57.44 will be considered in detail in the subsequent discussion.

**TABLE I.** Parameters of vents (ACH = 57.44).

Vent model	Flow direction	Number of vents	Vent dimensions (mm)	Flow velocity (m/s)
1	Inlet	1	260 × 120	4.99
	Outlet	1	260 × 120	Atmospheric pressure
2	Inlet	1	780 × 40	4.99
	Outlet	1	175 × 180	Atmospheric pressure
3	Inlet	6	260 × 20	4.99
	Outlet	1	175 × 180	Atmospheric pressure
4	Inlet	1	175 × 180	4.99
	Outlet	6	260 × 20	Atmospheric pressure
5	Inlet	1	2600 × 40	1.50
	Outlet	4	2600 × 20	Atmospheric pressure

### D. Mathematical model

The Euler–Lagrange approach in CFD is widely used for simulating air-particle flow with low particle concentration. The Eulerian continuum equations are solved for the airflow, while the Newton's equations for motion are solved for the trajectories of particles. The stochastic point process is adopted to describe the dispersed phase statistically, with suitable coupling with an Eulerian statistical representation of the carrier fluid.

More precisely, a two-way coupled, Euler–Lagrange model is employed.^38^ The governing principles of the continuous phase (air) are the continuity and 3D Navier–Stokes (N–S) equations for an incompressible isothermal fluid. The volume fraction of fluid, *α*, is included [Eq. (1)].^39^ Large-eddy simulation (LES) is utilized for providing information for the turbulent flows. All variables of the flow field, including the velocity $\bar{u}$ and the pressure $\bar{p}$, are treated with a filter of the mesh size, except for the subgrid-scale (SGS) stress tensor **τ**_*sgs*_ which is resolved by the local dynamic one-equation eddy viscosity SGS model,^40^

$$\begin{array}{c} \frac{\partial \alpha }{\partial t}+\nabla \cdot \left(\alpha \bar{u}\right)=\;0,\\ \frac{\partial \left(\alpha \bar{u}\right)}{\partial t}+\nabla \cdot (\alpha \bar{u}\bar{u})=-\alpha \nabla (\frac{\bar{p}}{\rho })+\nu \nabla \cdot \nabla \left(\alpha \bar{u}\right)+\nabla \cdot \left(\alpha \tau_{\mathrm{sgs}}\right)+\alpha g-\frac{1}{\rho }\sum_{N}f_{p}. \end{array}$$ (1)

In all the incompressible solvers provided by OpenFOAM, density *ρ* is included implicitly. The kinematic pressure $\bar{P}=\bar{p}/\rho$ (instead of the pressure $\bar{p}$) and the kinematic eddy viscosity (rather than the dynamic eddy viscosity) are solved. The SGS stress tensor is expressed in the form

$$\tau_{\mathrm{sgs}}=\bar{u_{i}u_{j}}-{\bar{u}}_{i}{\bar{u}}_{j}.$$ (2)

The solution scheme will be discussed in Sec. II E 1. The equations governing the motions of the discrete phase (particles) are given by

$$m_{p}\frac{du_{p}}{dt}=\sum_{N}f_{p},$$ (3)

$$\sum_{N}f_{p}=f_{\mathrm{gravity}}+f_{\mathrm{drag}}+f_{\mathrm{other}},$$ (4)

where *f_p_* represents the forces acting on the particles. These forces include gravity *f_gravity_*, the drag *f_drag_* between air and the particles, and other components *f_other_*, e.g., the Basset force, the Saffman lift force, and the force due to virtual mass.^41–43^ Here, we consider the gravity and the drag force only. Particles are assumed to be spherical, and the drag model of a sphere is employed.

### E. Numerical method

The present study utilizes the open-source CFD library, *OpenFoam*, to simulate airflow and particle transport. *MPPICFoam* is a transient solver provided in *OpenFoam* for the coupled transport of a single kinematic particle cloud including the effect of the volume fraction of particles on the continuous phase.^44^ A special two-way coupling method is adopted, which takes into account the collisions between particles and flows without resolving particle-particle interactions.^45^ The nose patches of passengers are set as velocity inlet BCs for all simulation cases, where the time traces of airflow velocity are pre-set using the *codedFixedValue* model. The mouth surfaces of infected passengers are set as velocity inlet boundary similar to the nose patches, but only for the cases of coughing and sneezing. The mouth surfaces of healthy passengers in the cough and sneeze simulation cases, as well as mouth patches of all passengers in the breath cases, are set as wall BCs.

The PIMPLE (merged PISO-SIMPLE) algorithm is applied to solve the filtered N–S equations [Eq. (1)]. It is a combination of the SIMPLE (semi-implicit method for pressure-linked equations) and PISO (pressure-implicit split-operator) schemes which facilitates the transient solutions at higher Courant numbers.^44^ The gradient, divergence, and Laplacian terms of the filtered N–S equations are discretized utilizing the second-order-accurate Gauss linear scheme. The first-order-accurate Euler scheme is used for the time derivative terms. Gauss linear scheme is the standard finite volume discretization of Gaussian integration that employs a linear interpolation scheme. The equations of velocity *u* and SGS turbulence kinetic energy (TKE) *k_sgs_* are solved utilizing *smoothSolver* with the symmetric Gauss–Seidel smoother. The pressure *p* and volume fraction *α* are solved by GAMG (generalized geometric-algebraic multi-grid) solver. The solution tolerances of SGS TKE *k_sgs_*, volume fraction *α*, and velocity *u* are set to be 10^−5^, and a tolerance of 10^−6^ is employed for the pressure *p*. The steady-state solution for single-phase flows in the elevator cabin is used to initialize the time evolution of the air-particle flows. All transient simulation cases adopt adjustable time steps (0.0001 ≤ Δ*t*≤ 0.01 s) which conform to the Courant–Friedrichs–Lewy (CFL) condition.^46^ The maximum Courant number of the computational domain is set to be 1. To assess the influence of time step size on the accuracy of the computations, a comparison of solutions at different time step increments is presented in Sec. II E 3.

#### 1. Turbulence model

Turbulent flow is modeled using LES. LES calculates the large, energy-carrying eddies explicitly while it models the small eddies through a SGS turbulence model. Employing the Boussinesq eddy-viscosity hypothesis, the SGS stress tensor is described by Eq. (5), where *δ_ij_* is the Kronecker delta.^47^ The isotropic part of the SGS stresses, *τ_kk_*, is not modeled but is added to the pressure term. Eddy viscosity SGS model using a modeled balance equation [Eq. (6)] is invoked to simulate the behavior of SGS TKE *k_sgs_*.^48^ The SGS eddy viscosity, *ν_sgs_*, is then calculated using the resolved scales [Eq. (7)],

$$\tau_{\mathrm{sgs}}=\nu_{\mathrm{sgs}}\left(\frac{\partial {\bar{u}}_{i}}{\partial x_{j}}+\frac{\partial {\bar{u}}_{j}}{\partial x_{i}}\right)+\frac{1}{3}\delta_{ij}\tau_{kk},$$ (5)

$$k_{\mathrm{sgs}}=\frac{1}{2}\tau_{kk}=\frac{1}{2}\left(\bar{u_{k}^{2}}-{\bar{u}}_{k}^{2}\right),\frac{\partial k_{\mathrm{sgs}}}{\partial t}+{\bar{u}}_{j}\frac{\partial k_{\mathrm{sgs}}}{\partial x_{j}}=-\tau_{\mathrm{sgs}}\frac{\partial {\bar{u}}_{i}}{\partial x_{j}}-C_{\varepsilon }\frac{k_{\mathrm{sgs}}^{3/2}}{\Delta_{f}}+\frac{\partial }{\partial x_{j}}\left(\nu_{\mathrm{sgs}}\frac{\partial k_{\mathrm{sgs}}}{\partial x_{j}}\right),$$ (6)

$$\nu_{\mathrm{sgs}}=C_{k}k_{\mathrm{sgs}}^{1/2}\Delta_{f},$$ (7)

where Δ_*f*_ = 2 (*V_cell_*)^1/3^ is the filter-size, and *C_k_* and *C_ε_* are dynamically determined using the method presented in earlier works in the literature.^49^

#### 2. Particle model

Particles are injected into the elevator cabin by the exhale airflow through the mouth or nose of the passengers, but only particles from the infected person will be traced. The concentration and velocity of particles are determined by the flow rate of the injecting patch. The *patchFlowRateInjection* model implemented in *OpenFOAM* is employed on the boundary surfaces of the mouth or nose of the infected passenger and injects a number of parcels. The value of particles per parcel is set to be 1. Three particle injection models are established for the selected respiratory activities, namely, breathing, coughing, and sneezing. The size distributions of the particles are implemented using the *generalDistribution* model. The respiratory particles are small liquid elements that are supposed to latch onto a solid surface readily. All surfaces of the elevator and the passengers are set as “stick” boundaries for particles, and the vents are set as escape boundaries for particles.

In turbulent flows, the fluctuating air velocity influences the dispersion motion of particles to varying degrees. The Stokes number, *S_t_*, is a dimensionless parameter characterizing the behavior of particles suspended in fluid flows. It can be used to estimate the effects of fluctuating flows.^50^ More precisely, the Stokes number is defined as the ratio of the characteristic time associated with the particle motions to the corresponding timescale of the flow.^51^ Mathematically, the formulation is

$$St=\frac{t_{p}}{t_{\mathrm{air}}},\;\;\;t_{p}=\frac{\rho_{p}d_{p}^{2}}{18\mu_{\mathrm{air}}},\;\;\;t_{\mathrm{air}}=\frac{L}{u_{\mathrm{air}}},$$

where *t_p_* is the relaxation or response time of the particle, *t_air_* is the characteristic timescale of jet flow,^52^ *u_air_* is the bulk velocity of jet flow, *L* is the average length of injecting patch, *ρ_p_* is the particle density, *d_p_* is the particle diameter, and *μ_air_* is the dynamic viscosity of air.

**FIG. 6.**
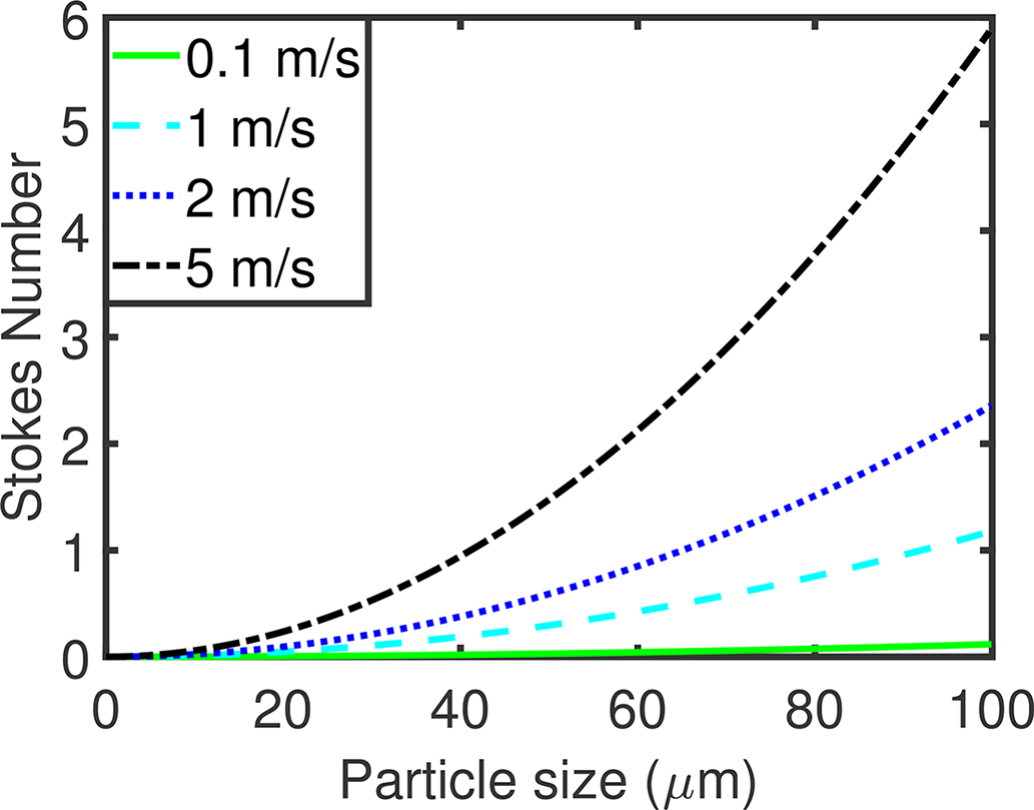
The dependence of the Stokes number on the particle size.

The typical length scale in this simulation is set to be 25 mm. The flow velocity varies from 0 to 5 m/s. The dependence of the Stokes number on the particle diameter for various wind speeds is illustrated in Fig. 6. In this study, the diameters of particles suspended in air are smaller than 100 μm, and the majority is actually below 10 μm. With the Stokes number being much less than 1, such particles will be driven along the fluid streamlines.^53^ The SGS dispersion of particles should be considered and will be accounted for by the stochastic dispersion model.

#### 3. Grid and time step independence study

**FIG. 7.**
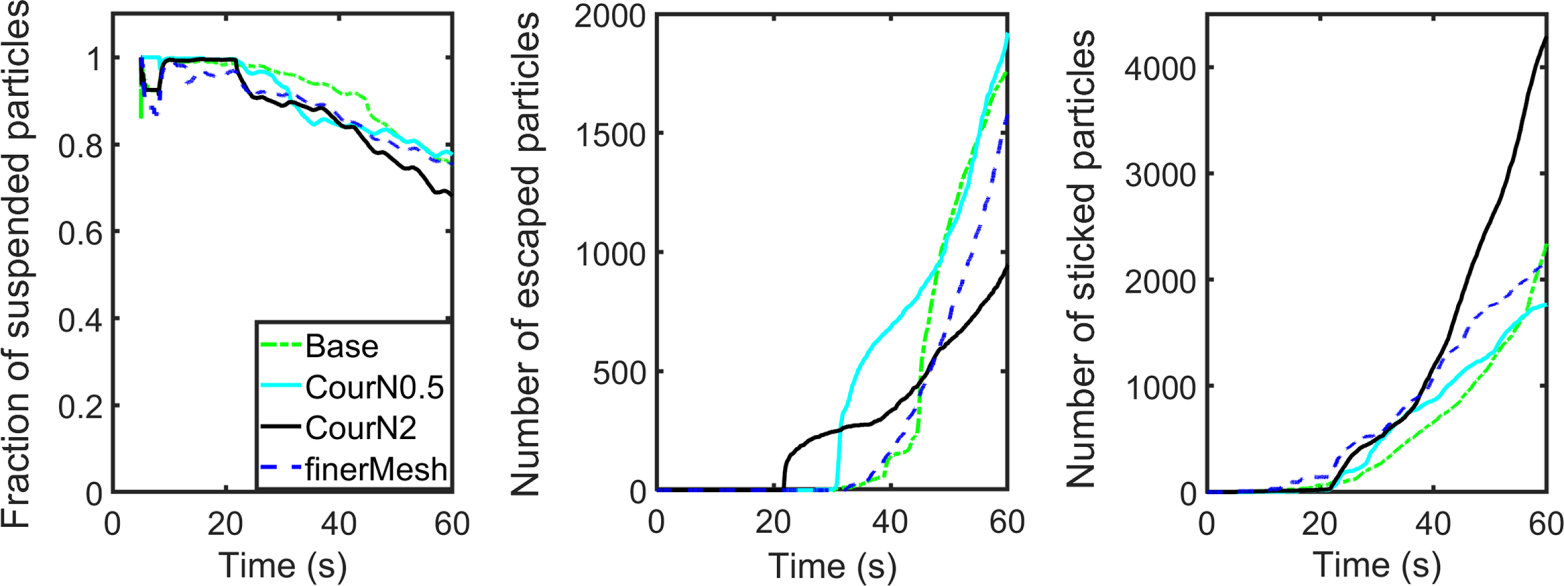
The dependence of grid and time step size.

To investigate the influence of grid size and time step increment, we select as a representative example the case of vent model 1 with an ACH of 57.44. Four simulations (one basic set, one for enhanced spatial resolution, and two for enhanced temporal resolution) on the dispersion of breath particles are carried out. For enhanced spatial resolution, finer meshes with 1 400 000 cells, with a minimum (maximum) cell size of 1.5 (20) mm, are used for a grid independence study. For the marching forward in time, two studies with larger and smaller time step increments which correspond to a maximum Courant number of 2 and 0.5 are conducted for quantifying the time step sensitivity. The simulation results of the four cases are compared and illustrated in Fig. 7. By comparing with the base case, the differences of the case using finer mesh and the case using smaller time step increments, in terms of qualitative trends and quantitative numbers, especially for the fraction rate of suspended particles, are relatively small. However, the result of the case using larger time step increments shows greater differences in comparison with the base case. The computational times of the four cases using a 10-core processor are 20, 51, 14, and 37 h, respectively. We believe that a balance between efficient computer usage and accuracy has been achieved with the present effort.

To further verify the capacity of the grid on resolving turbulent structures and to determine the optimal mesh in the present LES simulations, we employ the standard *k*-*ε* turbulence model to resolve the distribution of turbulent kinetic energy (*k*) and the rate of dissipation of turbulent kinetic energy (*ε*). The integral length scale, *l*_0_, which is the length of an eddy at the average kinematic energy of all the eddies, is calculated using the results of the *k*-*ε* turbulence model based on local equilibrium, namely, *l*_0_ = *k*^3/2^/*ε*.^54,55^

**FIG. 8.**
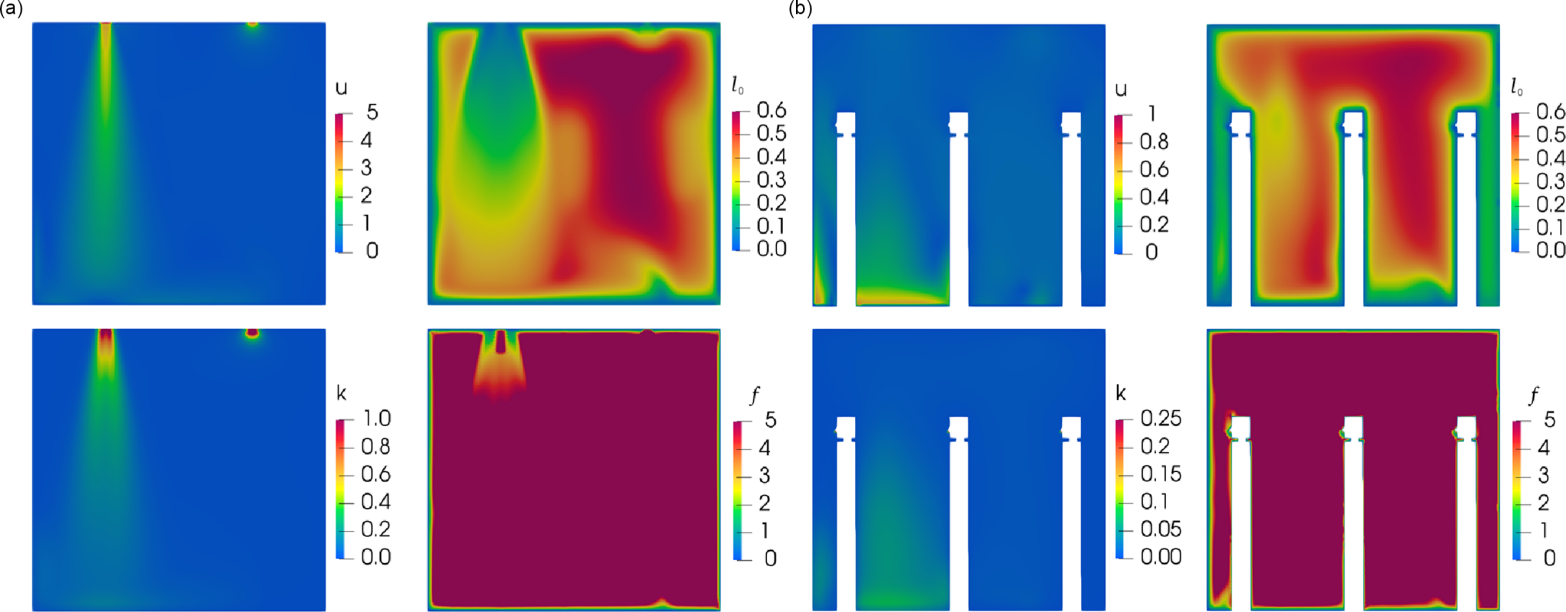
Contour plots of the flow velocity, integral length scale, kinematic turbulent energy, and parameter *f* on the planes of (a) *x* = 0.775 m and (b) *x* = 1.15 m at time = 8 s.

As the integral length scale represents the characteristic dimension of the eddies, a parameter, *f* = *l*_0_/Δ*x*, is defined as the ratio of the integral length scale *l*_0_ to the grid size Δ*x*,^56^ where Δ*x* = (*V_cell_*)^1/3^ is used to estimate the mesh resolution criterion for the LES. The recommended value of *f* is over 5.0, with the goal of 80% or more of the energy spectrum being well-resolved.^55,57,58^ The shaded contours of the velocity, integral length scale, kinematic turbulent energy, and *f* on the *y*–*z* plane at *x* = 0.775 and 1.15 are shown in Figs. 8(a) and 8(b), respectively. The minimum cell size of 1.75 mm and the maximum cell size of 28 mm are adequate in achieving the desired accuracy.^59,60^

### F. Case study

Three different factors that can influence the transport of virus have been studied, namely, respiratory activities of the infected person, the position of the infected person, and the ventilation system of the elevator. In terms of the ventilation system, the properties of ventilation mode, air delivery capacity, and vent configuration must be taken into account. All the simulation cases in this study and the choice of parameters are listed in Table II. The simulation cases are divided into five groups, investigating the effects of

• respiratory events,
• ventilation mode and capacity,
• injector position,
• vent schemes, and
• performance of optimized vent scheme.

**TABLE II.** List of scenarios tested in this study.

No.	Group	Ventilation mode	Air delivery (m^3^/h)	Vent scheme	Passenger position	Event
1						breath
2	A	Natural	0	vent model 1	P3	cough
3						sneeze
4						breath
5			110			cough
6	B	Forced		vent model 1	P3	sneeze
7						breath
8			560			cough
9						sneeze
10					P6	
11	C	Forced	560	vent model 2	P3	breath
12					P1	
12				vent model 2		
13				vent model 1		
14	D	Forced	560	vent model 3	P1	breath
15				vent model 4		
16				vent model 5		
16					P1	
17	E	Forced	560	vent model 5	P3	breath
18					P6	

The single trip running time of the elevator, the time needed from the closing of the door on the ground floor to the opening of the door on the destination floor, is taken to be one minute. All 18 simulation cases are designed to run for 60 s. To reduce the effects of the initial flow field and to avoid a sharp change in velocity at the boundaries of the inlet, the injection of particle starts at 5 s after simulations of the breath cases begin, and a corresponding value of 7.7 s for the cough and sneeze cases.

## III. RESULTS AND DISCUSSION

### A. The effect of respiratory events

**FIG. 9.**
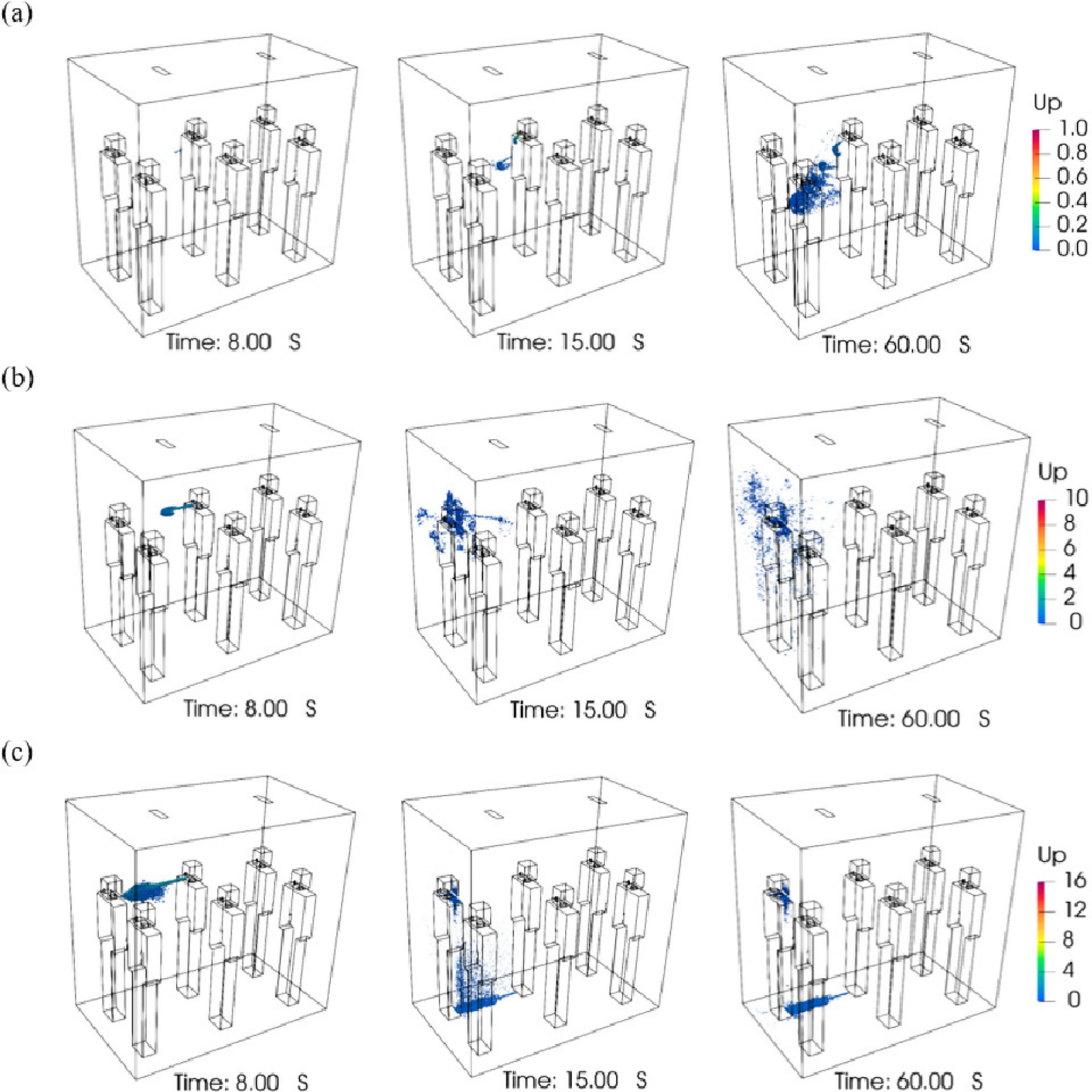
Particle distribution in the elevator, group A: (a) breath case, particle scale factor 10 000:1, (b) cough case, particle scale factor 2500:1, and (c) sneeze case, particle scale factor 100:1.

Human activities, e.g., breath, cough, or sneeze, will generate jet flows of different velocities which carry particles with a large range in concentration and size. To understand the transport and deposition of particles ejected by these respiratory activities, three cases have been simulated under natural ventilation, each with its own particle models and inlet velocity profiles. The particle distributions at the conclusion of the simulation are listed in Table III. To illustrate the particle dynamics in the elevator cabin, the distributions of particle positions as time evolves are shown in Fig. 9. Particle diameters of all three simulations are scaled up for easier visualization, with scale factors of 10 000:1, 2500:1, and 100:1.

**TABLE III.** Particle distributions of group A, time = 60 s.

No.	Group	Injected particles	Stuck particles (on wall)	Stuck particles (on person)	Escaped particles	Suspended particles (in air)
1		16 480	0	121	0	16 359
2	A	3 491	343	1272	0	1 876
3		11 700	7851	3789	0	60

In the breath case, all particles are smaller than 2-μm diameter. They diffuse forward and downward slowly as shown in Fig. 9(a). For the duration of the simulation, no particle escapes or adheres to the walls (Table III). Only 121 particles are stuck on the passengers. Almost all particles exhaled by breathing are still suspended in the air. In the cough case, particle sizes range from 1 to 10 μm. Less than half of the particles are deposited, and the rest are still suspended in the immediate neighborhood of the passenger standing in front of the coughing one. Among the deposited particles, most of them are stuck on the surfaces of the passenger in front of the coughing one [Fig. 9(b)]. In the sneeze case, almost all particles deposit quickly, while only a few small particles are still suspended. Some particles will stick on the body of the passenger in front of the sneezing one, and a greater portion of particles will stick on the floor between the two passengers [Fig. 9(c)].

**FIG. 10.**
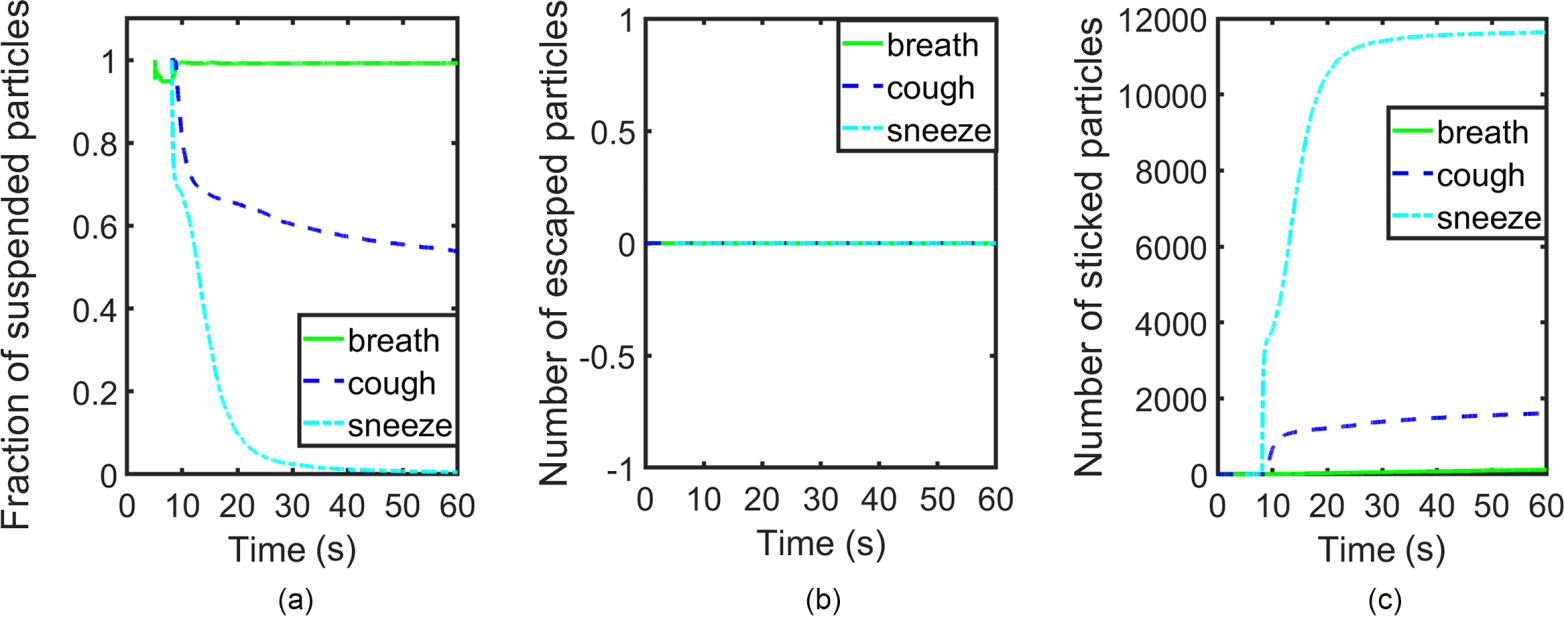
Results of group A: (a) number fraction of suspended particles, (b) number of escaped particles, and (c) number of stuck particles.

To quantitatively describe the processes of particle dispersion and deposition in each case, we plot the number of escaped particles, the number of stuck particles, as well as the ratio of suspended particles to total injected particles as functions of time (Fig. 10). We shall use an elevator ride of one minute as an illustrative example. For the breath case, as no particle escapes and only very few particles are deposited, the fraction of suspended particles is approximately unity most of the time. Hence, these sub-micrometer particles generated by breathing can persist in confined space for a significant portion of the duration of the elevator ride if there is no forced ventilation. As a comparison, the corresponding fractions of suspended particles in the cough and breath cases decrease to around 0.5 and 0, respectively. The fraction of suspended particles of cough reduces rapidly in the first 10 s after coughing, but then undergoes a much slower evolution for the remaining time. Hence, small particles ejected by coughing can be suspended for a long time. For the sneeze case, over 95% of the particles are deposited within the first 15 s from sneezing. The common feature of the three cases is that no particle will escape through the vents.

### B. The effect of ventilation mode and capacity

Most elevators are equipped with air fans or air conditioners for enhancing air circulation and improving air quality. In this study, two forced ventilation modes, with the ACH of 11.28 and 57.44, are studied. Breath, cough, and sneeze cases introduced in Sec. II B will be simulated under the two forced ventilation modes, i.e., a total of six cases. Table IV shows the particle distributions of the six scenarios. For comparison, the results of natural ventilation cases are analyzed together with the forced ventilation simulations. Regardless of the effects of ambient temperature and air convection outside the elevator, natural ventilation has been treated as a special forced mode with a ventilation capacity of 0 m^3^/h (ACH = 0).

**TABLE IV.** Particle distributions of group B, time = 60 s.

No.	Group	Injected particles	Stuck particles (on wall)	Stuck particles (on person)	Escaped particles	Suspended particles (in air)
4	B	16 474	1	171	0	16 302
5		3493	261	1132	0	2100
6		11 694	7950	3687	0	57
7		16 393	1761	577	1760	12 295
8		3487	1200	1192	159	936
9		11 675	6963	4631	8	73

In the breath cases, the numbers of escaped particles and stuck particles are barely changed when the ACH varies from 0 to 11.28, but then display an upward trend if the ACH increases from 11.28 to 57.44. Similar to the breath cases, the numbers of escaped particles of cough cases with ACHs of 0 or 11.28 are zero, and the numbers of both escaped particles and stuck particles go up when the ACH is increased to 57.44. In contrast with the breath cases, the number of stuck particles in the cough cases decreases as the ACH changes from 0 to 11.28. As a result, the number of suspended particles increases. However, the number of suspended particles of the sneeze cases remains almost the same. A shift with an order of magnitude of 100/1000 occurs in the numbers of particles adhered onto the passengers and walls when the ACH increases “from 0 to 11.28”/“from 11.28 to 57.44,” respectively. Furthermore, in contrast to the cough cases, most of the particles exhaled by sneezing adhere onto the walls instead of the passengers.

**FIG. 11.**
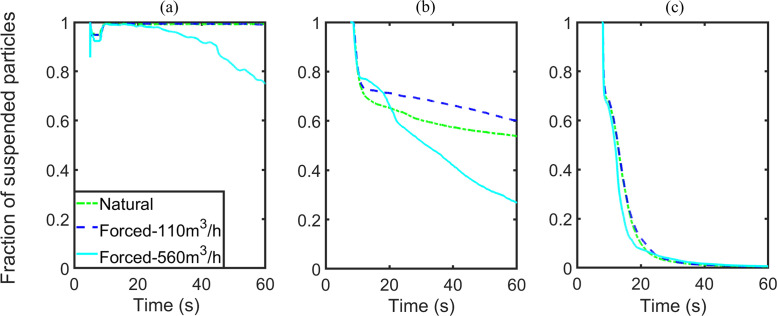
Number fraction of suspended particles, group B: (a) breath, (b) cough, and (c) sneeze case.

**FIG. 12.**
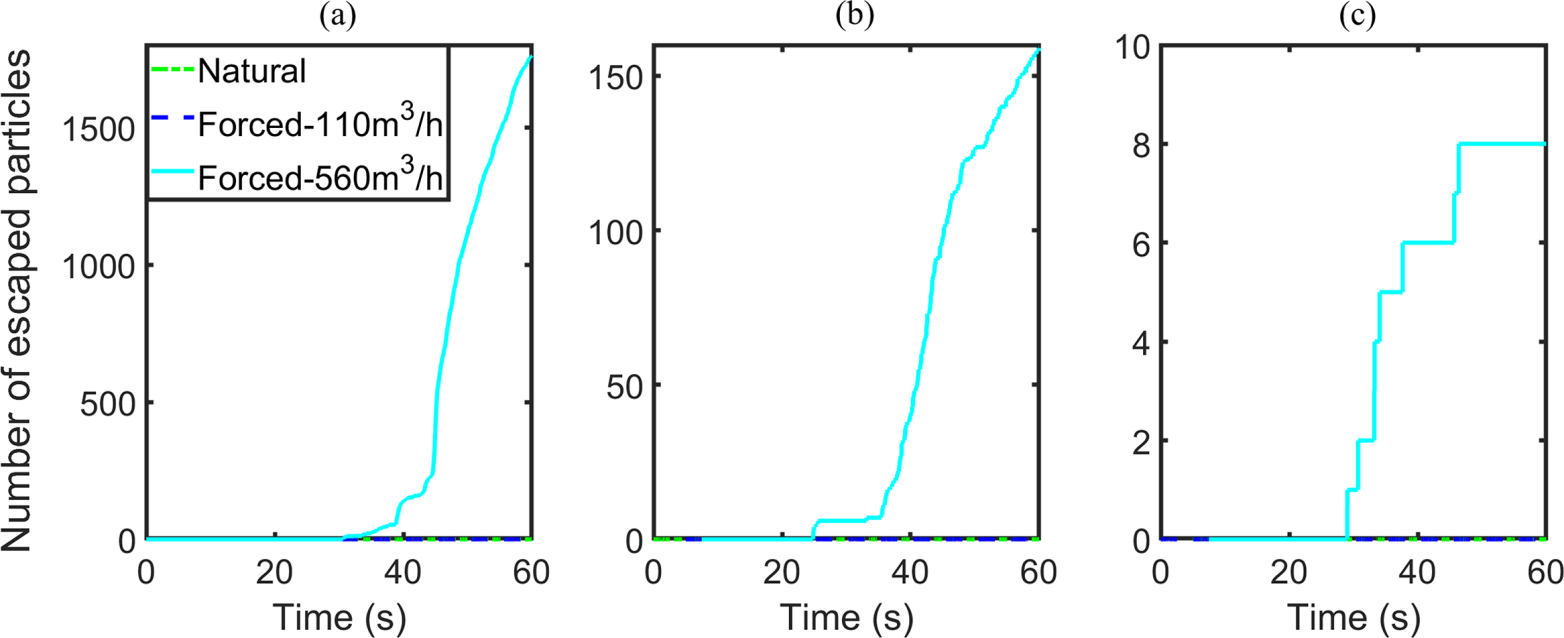
Number of escaped particles, group B: (a) breath, (b) cough, and (c) sneeze case.

**FIG. 13.**
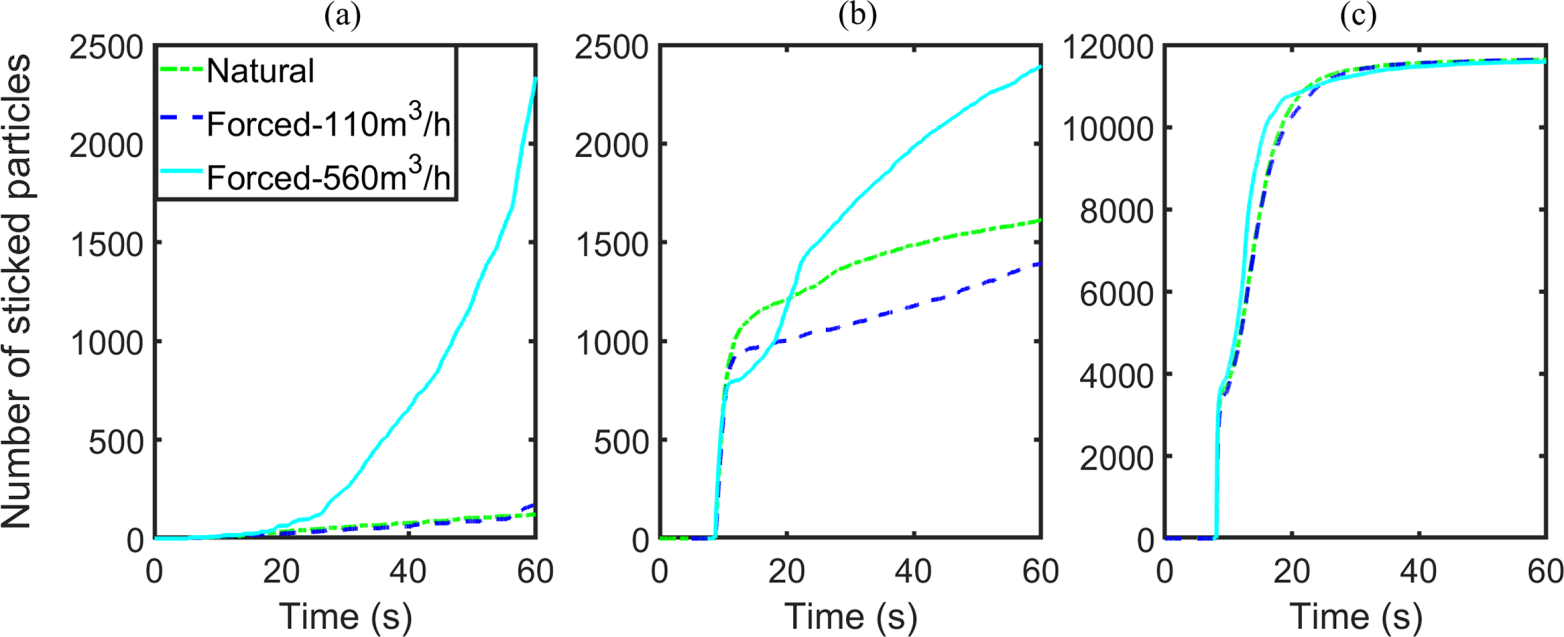
Number of stuck particles, group B: (a) breath, (b) cough, and (c) sneeze case.

Similarly, the variations in the numbers of escaped particles, adhered particles, and the number fraction of suspended particles to injected particles are illustrated in Figs. 11–13, respectively. By comparing the results for situations with ACH of 0 and 57.44, forced ventilation can accelerate the reduction of suspended particles for the breath and cough cases [Figs. 11(a) and 11(b)]. Significant changes are observed because the numbers of escaped particles and adhered particles both increase [Figs. 12(a), 12(b), 13(a), and 13(b)]. In the breath case with an ACH of 11.28, the forced airflow does not generate a significant difference in the numbers of escaped, stuck, and suspended particles. For cough cases, the influence of airflow on particles deposition is sensitive to the ventilation capacity. The number of stuck particles for ACH being 11.28 is even smaller than the value under natural ventilation. The underlying reason is that forced airflow slows down the deposition of most particles, which will otherwise be deposited immediately after coughing. The airflow decelerates the deposition speed of particles by generating a drag force with a vertical component to cancel out the effect of gravity. For both the breath and cough cases, if the ventilation capacity is large enough (ACH being 57.44), the strongly forced airflow will generate a large drag force that outstrips the effect of gravity. This force promotes the escape or deposition of particles and thus reduces the number of particles suspended in the air. The effect of forced airflow on the sneeze case is exceedingly small, where only a few particles can escape [Figs. 12(c) and 13(c)]. The velocity of particle deposition in the first 15 s after sneezing has accelerated, while the sedimentation of particles in the rest of the time has been slowed. In summary, the forced airflow

• enhances the motions of small particles,
• facilitates the deposition of micrometer or sub-micrometer particles,
• suppresses the deposition of particles with diameters in the range of 10–100 μm,
• does not pose a significant influence on the motions of particles much larger than 100 μm.

**FIG. 14.**
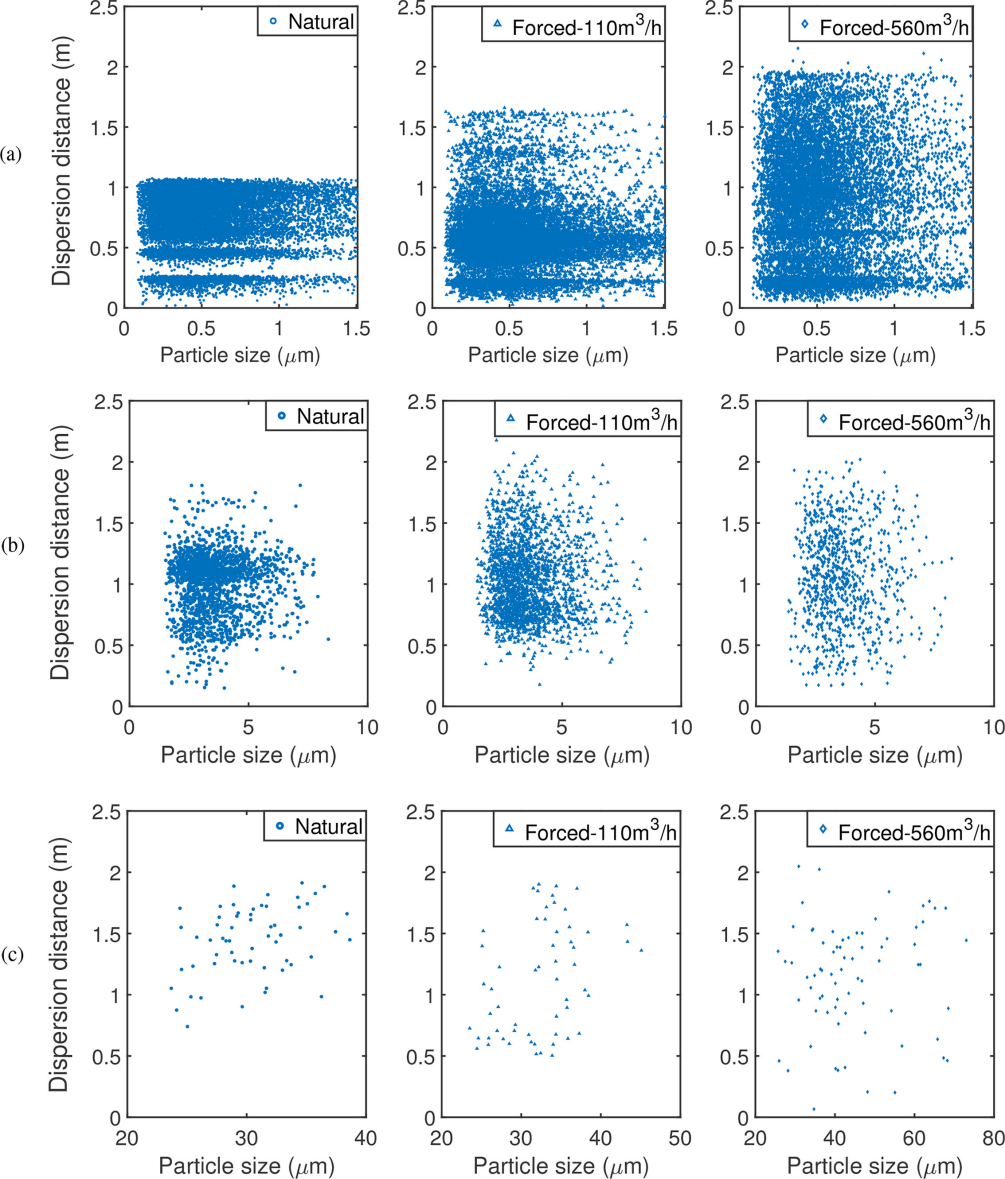
Dispersion distance of suspended particles, group B: (a) breath, (b) cough, and (c) sneeze case.

For a detailed analysis on the effects of ventilation mode on the movement of the particles, the dispersion distances of suspended particles at the conclusion of the simulation are measured (Fig. 14). In general, the dispersion distance of airborne particles increases with increasing ventilation capacity. Forced ventilation generates intensive air current inside the cabin and promotes the dispersion of particles in the breath, cough, and sneeze cases. The particles of the cough case disperse in very much the same way as the breath cases. In the sneeze cases, the sizes of suspended particles increase with ventilation capacity. The strong current carries particles that otherwise might have deposited onto solid surfaces into the air. As the effect of forced airflow on suspended particles of the cough and sneeze cases is relatively small, only the breath cases will be discussed in Secs. III C–III E.

### C. The effect of the position of infected passenger

The number of (normal and infected) passengers and their relative positions within an elevator may in general vary widely. We shall study one special case as an illustrative example. More precisely, we consider the configuration of six passengers standing in a two-row, rectangular pattern, and one passenger is infected. Three possible positions of the infected passenger are studied from the one nearest to the outlet vent to the one furthest from the outlet vent [positions P6, P3, and P1, Fig. 1(c)]. Particles injected near the outlet vent escape sooner than others, and the number of escaped particles decreases first from 3389 to 1247 and then to 356, as the distance between injector and outlet vent increases (Table V). There may be two underlying reasons. First, air circulation near the vent is well-organized and the “out flow” is stable. Second, the distances the particles need to travel to escape through the vent are relatively short. The number of particles adhered onto the passengers increases with the distance between the infected passenger and the outlet vent (from 354 to 627 and then 1256) as a longer traveling distance means that more particles will interact with the surfaces of the passengers. For the simulation cases with particle injectors near the walls (position P6 and P1), the number of particles stuck on the walls is more than the case with a particle injector in the middle of the elevator (position P3).

**TABLE V.** Particle distributions of group C, time = 60 s.

No.	Group	Injected particles	Stuck particles (on wall)	Stuck particles (on person)	Escaped particles	Suspended particles (in air)
10		16 919	1926	354	3389	11 250
11	C	16 625	1643	627	1247	13 108
12		16 638	2257	1256	356	12 769

**FIG. 15.**
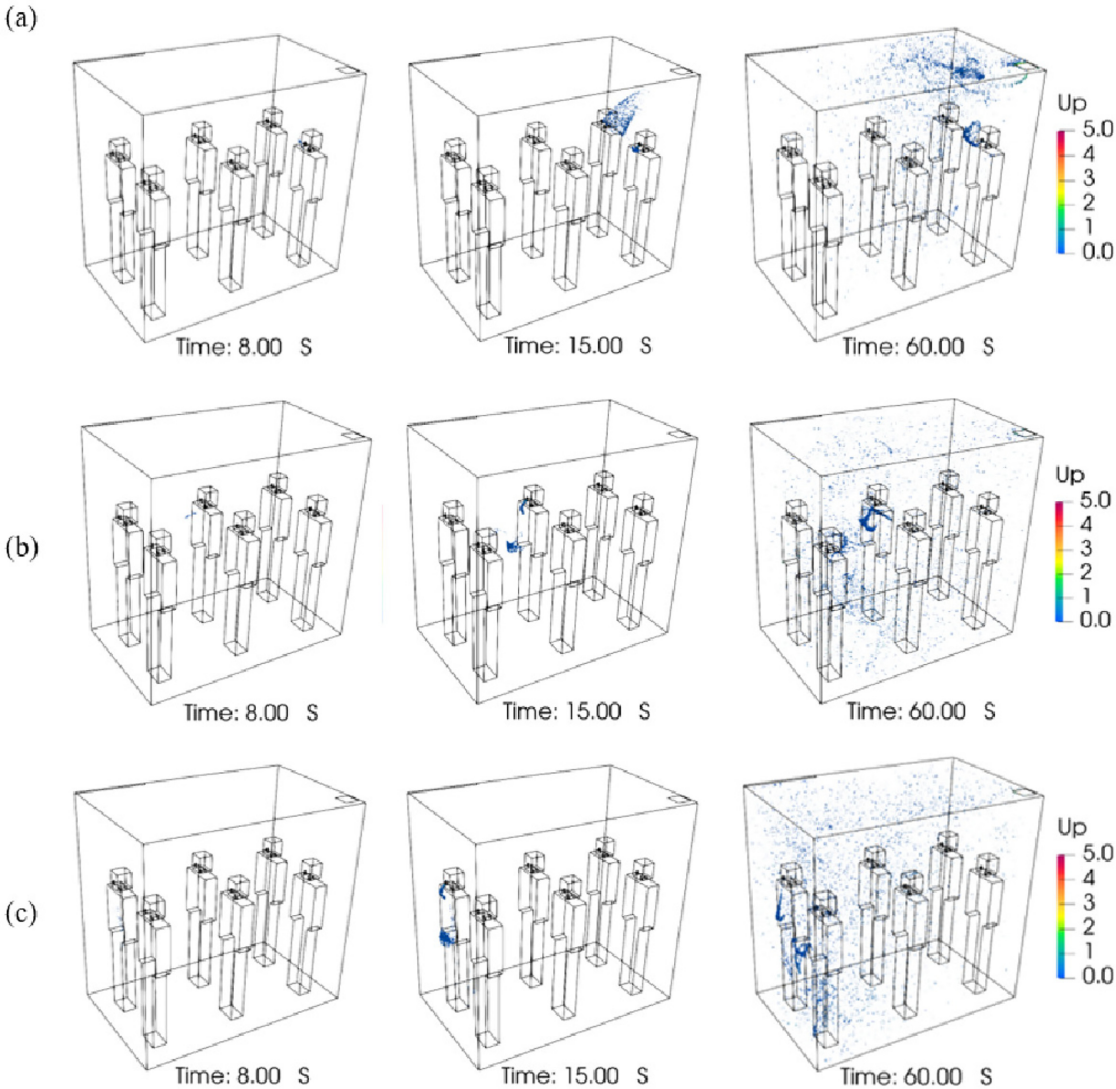
Particle distribution in the elevator, group C: (a) position P6, (b) position P3, and (c) position P1. Particle scale factor 1000:1.

The positions and distributions of particles in relation to the vents of the elevator cabin can be elucidated (Fig. 15). If the infected passenger is located at position P6, nearest to the outlet, particles are mainly located at the right segment of the cabin and only a small portion diffuses toward the left-hand side [Fig. 15(a)]. If the particle injector is set to be at the position P3 [Fig. 15(b)], in the middle of the lift cabin, particles tend to concentrate in the middle segment and disperse toward both left- and right-hand sides. Finally, if the infected passenger stands at position P1, the furthest from the outlet, particles will cluster at the right segment of the lift cabin and travel across the cabin toward the outlet.

**FIG. 16.**
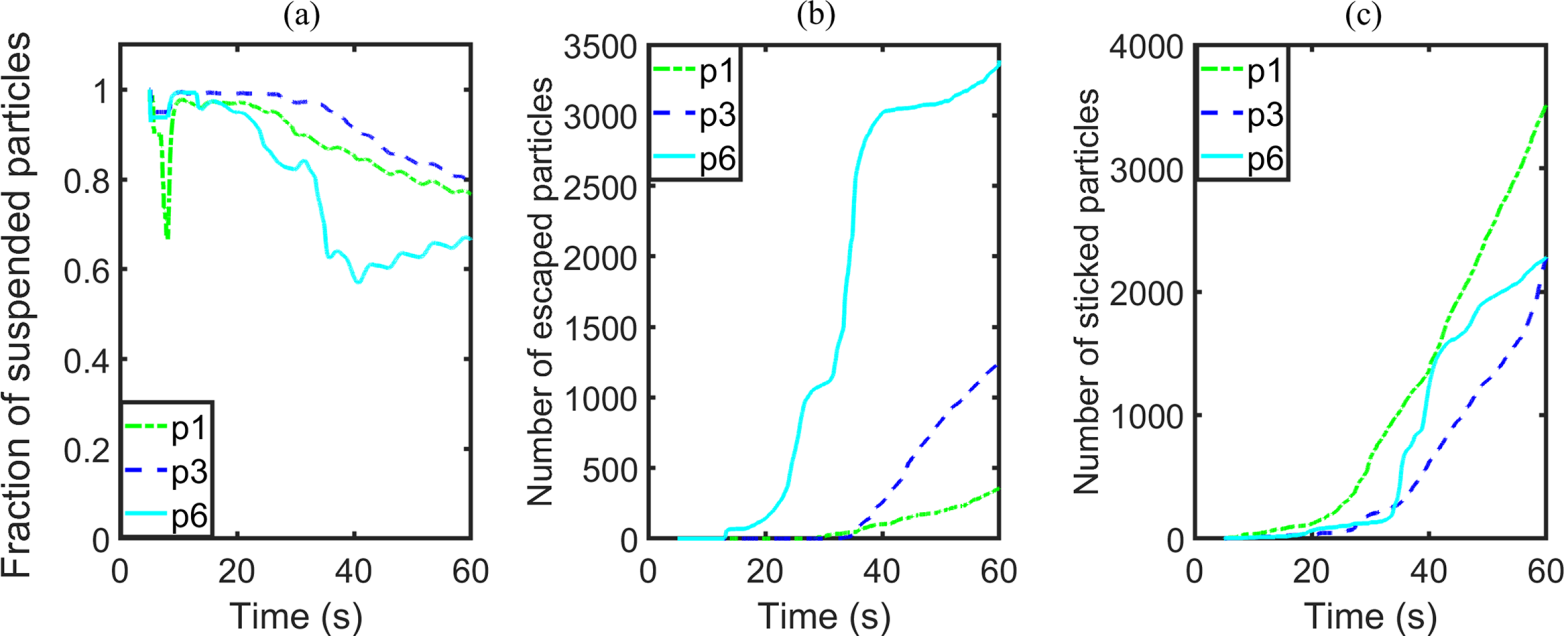
Results of group C: (a) number fraction of suspended particles, (b) number of escaped particles, and (c) number of stuck particles.

The effects of injector position on the changes of the numbers of escaped particles, stuck particles, and the ratio of suspended particles to injected particles are illustrated in Fig. 16. The number of escaped particles increases as the distance between injector position and outlet vent decreases. If the injector is at position P6 [Fig. 16(a)], the ratio of suspended particles to injected particles attains a minimum value of about 0.63 as the escaped particles increase significantly. The pattern for stuck particles in association with the position of the injector is not monotonic as this relation is also influenced by the position of the walls.

### D. The effect of vent schemes

To study the effect of vent schemes on the transmission of aerosol particles, three typical ceiling vent schemes and two vent models, which adopt both ceiling and sidewall vents, are studied. For all the vent schemes, position P1 is the furthest away from the outlet vent. Hence, the escape of particles injected from P1 will suffer the highest difficulty. Based on the previous results, ACH is set as 57.44 and we place the infected passenger at position P1 to minimize the influence of ventilation capacity and injector position. Table VI lists the particle distributions for all simulations considered. The three ceiling vent schemes exhibit poor performance with 12 769, 11 802, and 12 530 particles still suspended in the air. Vent model 4 and vent model 5 display a much better performance by including one inlet located at the center of the ceiling and several outlets at the bottom of the sidewalls. This kind of vent scheme helps to improve the flow field and is thus beneficial for the escape and deposition of particles. For example, only 3445 particles are airborne for vent model 5 at the end time of the simulation.

**TABLE VI.** Particle distributions of group D, time = 60 s.

No.	Group	Injected particles	Stuck particles (on wall)	Stuck particles (on person)	Escaped particles	Suspended particles (in air)
12		16 638	2257	1256	356	12 769
13		16 480	3180	1242	256	11 802
14	D	16 215	2479	802	404	12 530
15		16 397	6338	1644	1212	7203
16		15 880	7142	2378	2915	3445

**FIG. 17.**
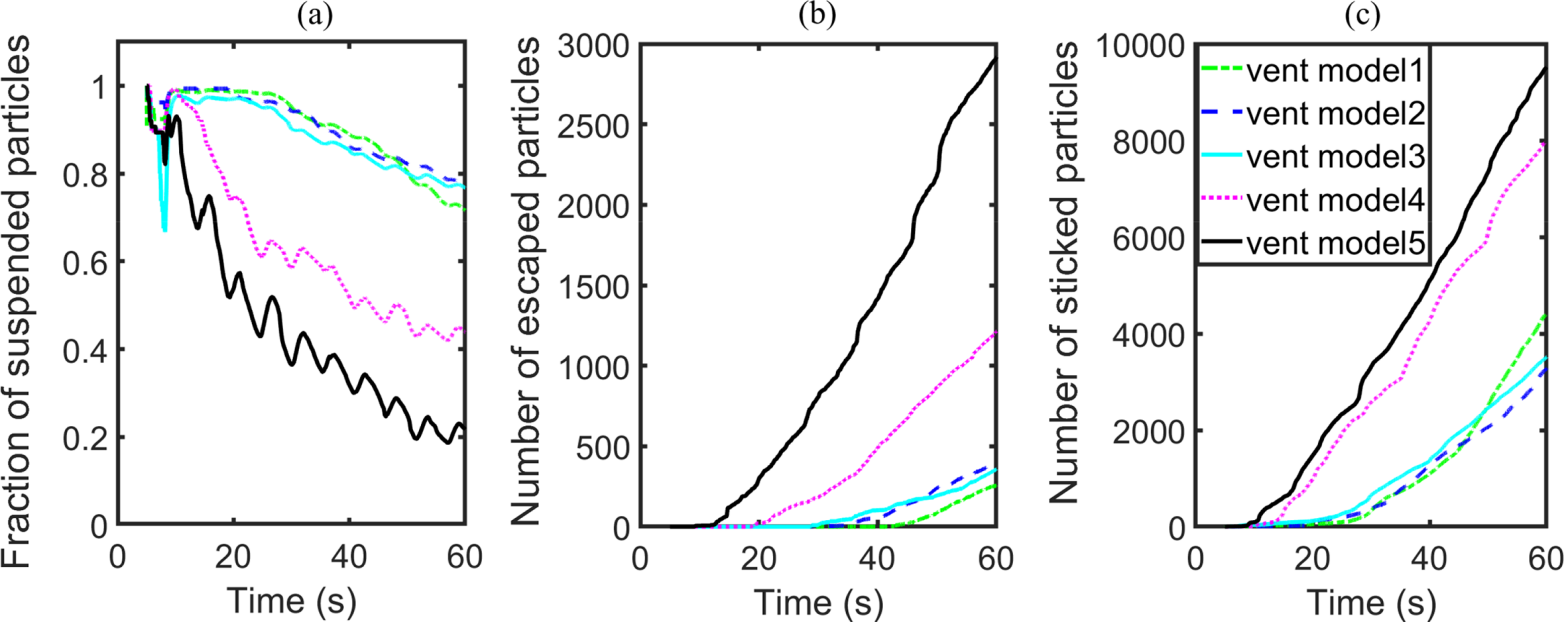
Results of group D: (a) number fraction of suspended particles, (b) number of escaped particles, and (c) number of stuck particles.

The actual quantitative variations in terms of numbers of particles are illustrated in Fig. 17. The difference in the performance of the three ceiling vent schemes is not significant, in spite of the relatively large difference in the vent configurations. In contrast, the two vent models with combinations of ceiling and sidewall vents both facilitate the deposition as well as escape of particles, and reduce the number of suspended particles in the air effectively.

### E. The performance of the optimized vent scheme

According to the results of groups C and D, the position of the particle injector plays a significant influence on the spreading of particles. As vent model 5 enhances the elimination of particles inside the elevator cabin effectively, it is selected to study the performance of the optimized vent scheme in terms of the effects of particle injector position. We choose ACH to be 57.44, and assign the infected passengers to stand at positions P1, P3 or P6. Two indicators show visible variations for the three positions, namely, particles suspended in air and particles stuck on passengers (Table VII). Particles in the air can vary from a minimum of 2414 to 3785, and particles stuck on passengers vary from 2378 to 3977. In contrast, the numbers of escaped particles and particles adhered onto the wall show only much smaller variations, with the minimal and maximal values only differing by 5.5% and 6.3%, respectively. To sum up, there is an 8% differential in the fractions of suspended particles.

**TABLE VII.** Particle distributions of group E, time = 60 s.

No.	Group	Injected particles	Stuck particles (on wall)	Stuck particles (on person)	Escaped particles	Suspended particles (in air)
16		15 880	7142	2378	2915	3445
17	E	16 846	7465	2575	3021	3785
18		16 848	7594	3977	2863	2414

**FIG. 18.**
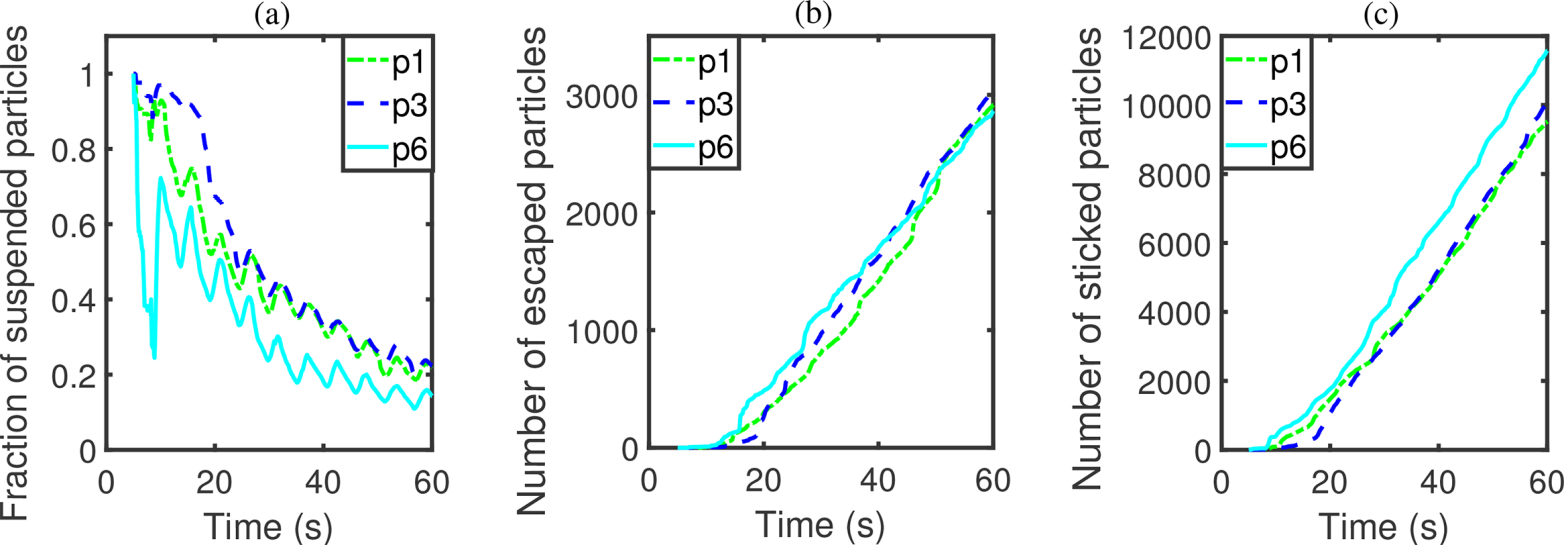
Results of group E: (a) number fraction of suspended particles, (b) number of escaped particles, and (c) number of stuck particles.

The difference between stuck particles among the three cases increases with time (Fig. 18). Eventually, the fractions of suspended particles all approach asymptotically to a constant value of about 0.2. This scenario will represent an adequate performance in the elimination of suspended particles. Hence an optimized vent model will remove any possible deterioration of performance induced by varied injector positions.

## IV. CONCLUSIONS

The spread of the aerosol potentially carrying pathogen in an elevator cabin has been investigated. Effects of three factors, namely, human respiratory events, locations of the infected person, and ventilation system are quantified. Different respiratory activities, e.g., breath, cough, and sneeze, are defined through the size distributions of the exhaled particles and distinct velocities of the expiratory airflows. Three particle injector positions (i.e., three possible locations of the infected passenger), ordered according to the distance to the outlet, are studied numerically in terms of particle removal. Three aspects of the ventilation system are considered, including ventilation mode, ventilation capacity, and vent schemes. More precisely, two ventilation modes (natural and forced ventilation) and two forced ventilation capacities (110 and 560 m^3^/h) are studied. Furthermore, the performances of particle removal of five vent schemes, including four commonly used ones and an optimized vent scheme, are assessed.

The particle motions in natural ventilation mode and two forced ventilation cases are compared. Most particles exhaled by sneezing and a portion (20%–30%) of the particles ejected by coughing tend to deposit on walls or other surfaces rapidly (in the first 10–15 s after sneezing or coughing). In contrast, a small portion of the particles in the sneeze case, and about 10%–50% of the particles of the cough case deposit slowly. Finally, aerosol particles generated by breathing are prone to be suspended in the air for a long period of time and will only disperse slowly. Forced air circulation may enhance or suppress the suspending of virus in the air, as strong airflow may generate drag forces which may strengthen or cancel out the effect of gravity. Hence larger particles, which may have otherwise deposited on solid surfaces, are carried into the air. Similarly, motions of small particles are accelerated, facilitating the deposition process of micrometer or sub-micrometer particles.

We assume that the risk of infection can be equated to the concentration of suspended/stuck particles of the specific respiratory events. For the sneeze case, the passenger standing in front of the injector person suffers the highest probability of infection. For the cough case, the particles will increase the risk of infection for all the people surrounding the infected passenger. For the breath case, the motion of particles can generally elevate the risk of infection throughout the confined space. The distances of the infected passenger to the outlet vent and the sidewalls will influence the dispersion and deposition of virus-laden particles. A shorter distance to the outlet is beneficial for the escape of particles, while a shorter distance to the sidewall facilitates the deposition of particles. Similar studies on airflows in elevators have been performed in the literature recently. A remark on comparisons will be in order. First, we treat configurations with multiple passengers instead of one single person.^21^ For another effort with three individuals (in contrast with the present case of six passengers),^20^ there are similarities (in terms of cabin volume and air flow rates) but also important differences. More precisely, a much wider range of particle sizes adopted here allows modeling of various respiratory events. In terms of fluid physics, LES are used here instead of the Reynolds-averaged turbulence schemes.

From our numerical simulations, a proper combination of vent models at the ceiling and sidewall vents displays a better performance than that of the three commonly used ceiling vent schemes, in terms of preventing the transport of particles carrying the SARS-CoV-2 virus. As an illustrative example on the performance of an optimized vent scheme (named vent model 5 in Sec. III), the fraction of suspended particles in the air can be reduced by as much as 80%. The application of this optimized vent model also exhibits similar purification functions numerically as we vary the locations of the infected passenger. These results will highlight the importance of a ventilation system in preventing the spread of the virus. Experimental verifications of these simulations are currently being carried out and will be reported in a future publication. Our findings on the effects of ventilation system are particularly true for elevators but not necessarily so for other confined spaces where separation distances of the majority of occupants from the index patient are much longer, e.g., airplane cabins, buses and classrooms. The increased dispersal of large particles (≥20 μm) due to air conditioning would be less important to overall aerosol transmission in such enclosed environments. We hope that the approach used and the insight gained in this study will be illuminating, e.g., in the settings of buses, classrooms and conference rooms.^61–63^ Other effects like Brownian motion might be needed in future studies.^64^ Hopefully more researchers will conduct similar investigations for crowded public places as we battle the SARS-CoV-2 virus.

## Data Availability

The data that support the findings of this study are available from the corresponding author upon reasonable request.
